# Automated left ventricular diastolic function evaluation from phase-contrast cardiovascular magnetic resonance and comparison with Doppler echocardiography

**DOI:** 10.1186/1532-429X-12-63

**Published:** 2010-11-09

**Authors:** Emilie Bollache, Alban Redheuil, Stéphanie Clément-Guinaudeau, Carine Defrance, Ludivine Perdrix, Magalie Ladouceur, Muriel Lefort, Alain De Cesare, Alain Herment, Benoît Diebold, Elie Mousseaux, Nadjia Kachenoura

**Affiliations:** 1 INSERM U678/UPMC Univ Paris 6, 91 Bd de l'Hôpital, 75013 Paris, France; 2 Radiology department, APHP, European Hospital Georges Pompidou, 20 rue Leblanc, 75015 Paris, France; 3 Echocardiography department, APHP, European Hospital Georges Pompidou, 20 rue Leblanc, 75015 Paris, France

## Abstract

**Background:**

Early detection of diastolic dysfunction is crucial for patients with incipient heart failure. Although this evaluation could be performed from phase-contrast (PC) cardiovascular magnetic resonance (CMR) data, its usefulness in clinical routine is not yet established, mainly because the interpretation of such data remains mostly based on manual post-processing. Accordingly, our goal was to develop a robust process to automatically estimate velocity and flow rate-related diastolic parameters from PC-CMR data and to test the consistency of these parameters against echocardiography as well as their ability to characterize left ventricular (LV) diastolic dysfunction.

**Results:**

We studied 35 controls and 18 patients with severe aortic valve stenosis and preserved LV ejection fraction who had PC-CMR and Doppler echocardiography exams on the same day. PC-CMR mitral flow and myocardial velocity data were analyzed using custom software for semi-automated extraction of diastolic parameters. Inter-operator reproducibility of flow pattern segmentation and functional parameters was assessed on a sub-group of 30 subjects. The mean percentage of overlap between the transmitral flow segmentations performed by two independent operators was 99.7 ± 1.6%, resulting in a small variability (<1.96 ± 2.95%) in functional parameter measurement. For maximal myocardial longitudinal velocities, the inter-operator variability was 4.25 ± 5.89%. The MR diastolic parameters varied significantly in patients as opposed to controls (p < 0.0002). Both velocity and flow rate diastolic parameters were consistent with echocardiographic values (r > 0.71) and receiver operating characteristic (ROC) analysis revealed their ability to separate patients from controls, with sensitivity > 0.80, specificity > 0.80 and accuracy > 0.85. Slight superiority in terms of correlation with echocardiography (r = 0.81) and accuracy to detect LV abnormalities (sensitivity > 0.83, specificity > 0.91 and accuracy > 0.89) was found for the PC-CMR flow-rate related parameters.

**Conclusions:**

A fast and reproducible technique for flow and myocardial PC-CMR data analysis was successfully used on controls and patients to extract consistent velocity-related diastolic parameters, as well as flow rate-related parameters. This technique provides a valuable addition to established CMR tools in the evaluation and the management of patients with diastolic dysfunction.

## Background

Altered diastolic function, which is strongly related to the quality of left ventricular (LV) filling, is a source of heart failure: it has been shown that 40 to 50% of patients suffering from heart failure have a normal LV ejection fraction while their diastolic function is impaired [1]. Furthermore, diastolic impairment without global systolic dysfunction is related to poor outcome [2,3]. Thus, the early and robust detection and quantification of diastolic dysfunction is crucial for optimal patient management. In clinical routine, the evaluation of diastolic function is achieved using Doppler echocardiography [4]. More specifically, several conventional diastolic parameters are estimated: the early and late filling peak velocities of the transmitral flow (E and A) and E-wave deceleration time (DT), as well as the annular myocardial early longitudinal peak velocity (E'). It has been shown that the calculated ratios E/A and E/E', as well as DT, have a high prognostic value [4,5].

Cardiovascular magnetic resonance (CMR) with its recent developments in velocity encoding is increasingly used for the analysis of through-plane blood flows and myocardial velocities. Furthermore, several studies demonstrated the usefulness of phase-contrast (PC) CMR in the measurement of some of the aforementioned conventional diastolic parameters [6-8]. However, these analyses were mostly based on manual positioning of regions of interest (ROIs) within the transmitral flow area or the myocardium on multiple phases [8-15]. This manual positioning of ROIs is time-consuming and operator-dependent.

Accordingly, our first goal was to develop a robust technique to automatically delineate the transmitral flow pattern, as well as the myocardium throughout the cardiac cycle, and to extract functional diastolic parameters from both velocity and flow rate curves. Our second aim was to test the consistency of these parameters on a group of 53 subjects including controls and patients, by evaluating: 1) the correlation between CMR parameters and the echocardiographic indices acquired on the same day, and 2) the ability of both CMR and echocardiographic diastolic parameters to characterize LV dysfunction in patients with severe aortic valve stenosis, in which changes in diastolic parameters have been previously shown [16]. Moreover, the inter-operator variability of the CMR measurements was evaluated on a sub-group of 30 subjects.

## Methods

### Study population and acquisition protocols

A group of 53 subjects had an echocardiographic exam for the evaluation of LV function and a CMR exam on the same day. This group included 35 controls free from overt cardiovascular disease and 18 patients with severe aortic valve stenosis.

Subjects characteristics and clinical data of LV function and remodeling are summarized in Table 1, for both groups. The study protocol was approved by the institutional review board and informed consent was obtained from all participants.

**Table 1:** Controls and patients clinical characteristics

	Controls (35)	Patients (18)	p value
Age (years)	38 ± 16	75 ± 13	<0.0001
Gender	14 ♂/21 ♀	8 ♂/10 ♀	
**Echocardiographic measurements**			
			
Ejection fraction (%)	65 ± 6	66 ± 7	0.52
End-diastolic diameter (mm)	47 ± 4	45 ± 6	0.15
End-systolic diameter (mm)	31 ± 5	27 ± 6	0.01
Diastolic septal thickness (mm)	8.2 ± 1.3	12.2 ± 2.7	<0.0001
Diastolic posterior wall thickness (mm)	8.3 ± 1.1	11.2 ± 2.3	<0.0001
Lateral mitral annulus systolic (S') velocity (cm/s)	11.3 ± 2.2	8.0 ± 2.1	<0.0001
Aortic valve area/Body surface area (cm^2^/m^2^)		0.51 ± 0.15	
Mean aortic gradient (mmHg)		51 ± 20	

**CMR measurements**			
			
Ejection fraction (%)	64 ± 5	67 ± 14	0.06
End-diastolic Volume (ml)	128 ± 33	101 ± 28	0.0009
End-systolic Volume (ml)	46 ± 13	35 ± 19	0.004
LV mass/End-diastolic Volume (g/ml)	0.96 ± 0.25	1.61 ± 0.55	<0.0001
LV mass/Body surface area (g/m^2^)	65 ± 10	89 ± 30	0.0002

Echocardiographic and CMR measurements obtained for both controls and patients groups as well as the statistical significance of the differences in these values between the two groups

Doppler echocardiography was performed by an experienced echocardiographer ("top" ASE level) using a GEMS Vivid 7 system. Transmitral flow and mitral annulus longitudinal velocities were recorded during relaxed end-of-expiration with the patient lying in supine left lateral decubitus. The transmitral early filling and atrial filling peaks (E_US _and A_US_) velocities and deceleration time (DT_US_), as well as the lateral annular early peak (E'_US_) longitudinal velocity were measured. All recordings were performed with simultaneous electrocardiographic (ECG) recording.

CMR imaging was performed using a 1.5 T MRI system (Signa HDx, GEMS, Waukesha, WI, USA). Previously acquired 2-chamber and 4-chamber views allowed positioning of a retrospectively ECG-gated PC pulse sequences, in a plane perpendicular to the transmitral inflow and located below the mitral annulus at the level of the tips of the opened mitral leaflets. At this location, two dynamic PC series, corresponding to an entire cardiac cycle, were acquired during breathhold: 1) the transmitral flow velocity sequence (encoding velocity Venc = 180 cm/s, echo time TE = 3.1 ms, repetition time TR = 7.6 ms, views per segment = 2, view sharing was used resulting in an effective temporal resolution of 15 ms), and 2) a myocardial longitudinal velocity sequence (Venc = 15 or 20 cm/sec, TE = 5 ms, TR = 9.5 ms, views per segment = 2, view sharing was used resulting in an effective temporal resolution of 20 ms). For both sequences, the following parameters were used: flip angle = 20°, slice thickness = 8 mm, pixel spacing = 1.9 × 1.9 mm, matrix 256 × 128. To minimize background offsets and so that acquisition duration remained compatible with breath holding, a 50% rectangular field of view was used. The mitral annulus was always at the centre of the acquired image and away from the PE-wraparound.

**Figure 1:**
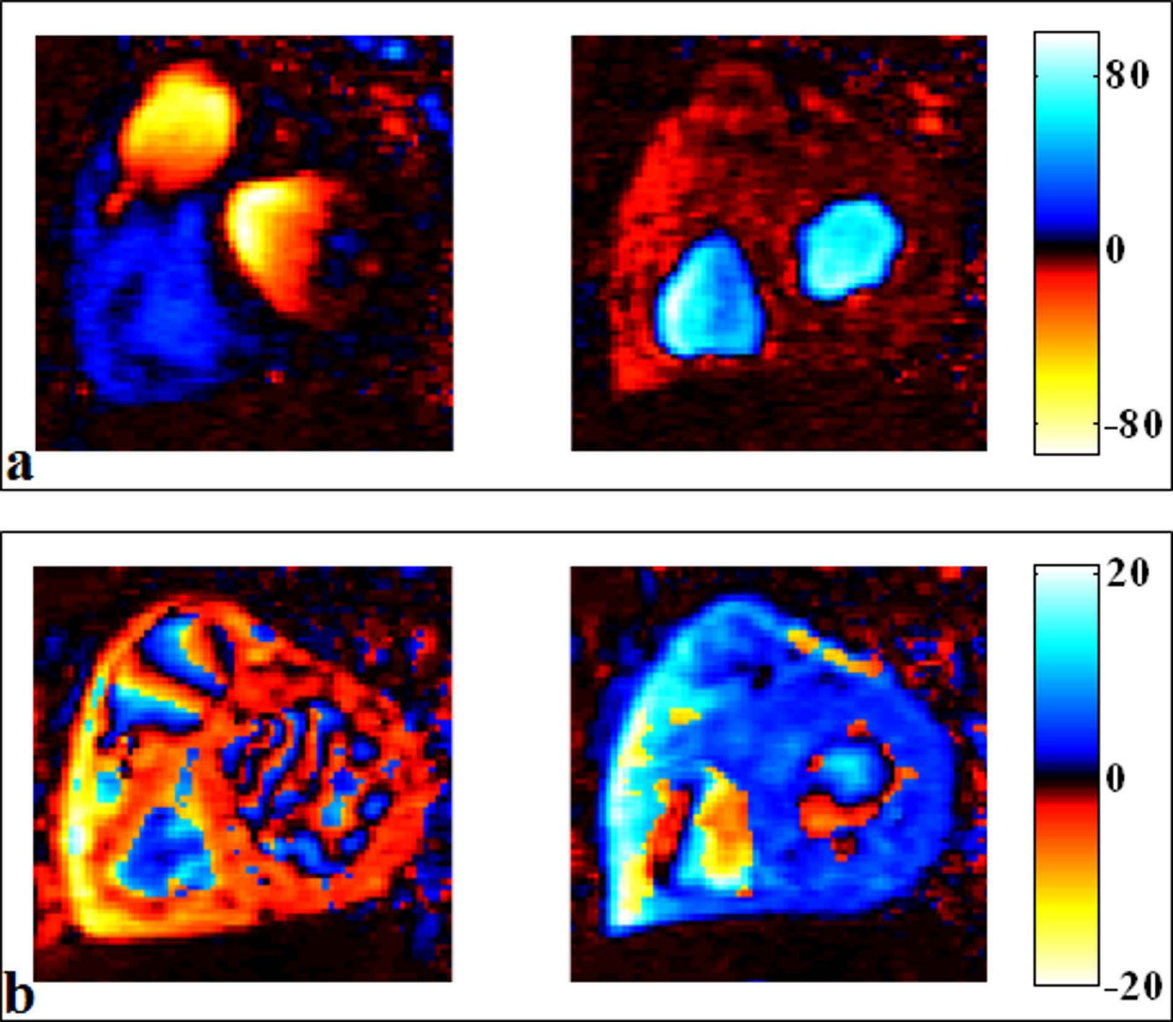
**Colour-coded display of the blood flow and myocardial longitudinal velocity-encoded PC images**. Panel a: blood flow velocity images, selected during a systolic phase (left), in which we can visualize the aortic ejection flow, and a diastolic phase (right), in which we can visualize the transmitral filling flow. Panel b: myocardial longitudinal velocity images, selected at the beginning of the systolic phase (left) and at the beginning of the diastolic phase (right). Negative velocity values were colour-coded in hot tones while positive velocity values were colour-coded in cold tones, to distinguish between through plane velocities in both directions.

Blood flow and myocardial velocity PC images were transferred for off-line analysis using a custom software. This software allowed a display of velocity images using an adapted colour scale designed to distinguish through-plane velocities in both directions (Figure 1). Our software included algorithms for blood flow and tissue delineation, as well as velocity and flow rate curves analysis.

### Semi-automated segmentation of blood flow velocity images

**Figure 2:**
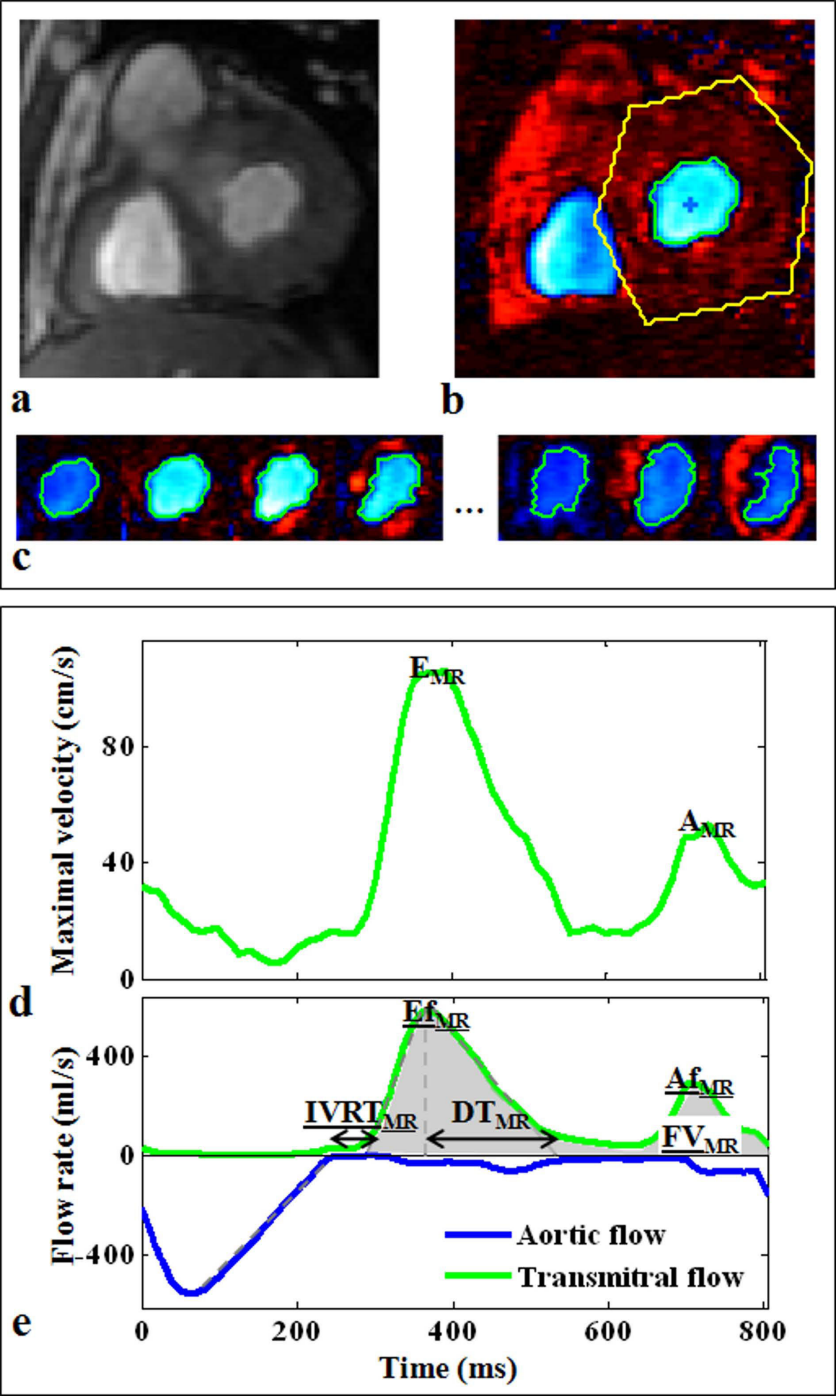
**Example of segmentation and diastolic parameters extraction from a transmitral flow PC dataset**. Top: description of the segmentation process performed semi-automatically on a velocity-encoded image after manual drawing of a rough region of interest around the transmitral flow (b). This segmentation resulted in a robust delineation of the transmitral flow pattern on each cardiac phase, as shown on the few selected phases (c) (see additional file 1: flowVideo, video file corresponding to the whole cardiac cycle). Of note, the modulus image corresponding to the phase image (b) was shown (a) to highlight the difficulty of segmenting such images. Bottom: the parameters automated extraction from the transmitral flow maximal velocity curve (d), and the transmitral (green) as well as the aortic (blue) flow rate curves (e), using the above segmentation. The estimated diastolic parameters (E_MR_, A_MR_, DT_MR_, IVRT_MR_, Ef_MR_, Af_MR_, FV_MR_) are indicated on velocity (d) and flow rate (e) curves.

Each PC dataset included a modulus dynamic series (Figure 2a) and the associated velocity-encoded dynamic series (Figure 2b), acquired during an entire cardiac cycle. The modulus images described the variation in the geometry of the mitral valve orifice during the cardiac cycle. These images were difficult to segment because of the flow-related contrast variations during the cardiac cycle, as well as the variable shapes of the mitral orifice. We therefore preferred to process velocity images, which presented connected areas in terms of pixel sign, defined by the local direction of the blood flow velocity.

Based on these connectivity properties, our segmentation algorithm comprised three main steps. First, a rough ROI was manually drawn on a single phase around the flow of interest (Figure 2b). This cardiac phase was automatically set to the middle of the cardiac cycle for transmitral flow segmentation and to the beginning of the cardiac cycle for aortic flow segmentation. The mean velocity curve was calculated within this ROI, and the cardiac phase corresponding to its highest absolute value was detected. In the second step, this latter cardiac phase was used to initialize the segmentation algorithm, by an automated detection of the biggest connected area, in terms of sign. The centre of mass of this area was calculated and reported on the neighbouring phases. In the third step, the biggest connected areas containing this centre of mass were detected on these neighbouring phases, and their centres of mass were used to repeat the process toward the beginning and the end of the cardiac cycle. The propagation of the centre of mass, while looking for the biggest connected area, constrained the segmentation process to track the flow of interest. This step provided a refined delineation of the blood flow pattern in each phase of the PC velocity series (Figure 2c).

### Blood flow functional parameters

After transmitral orifice segmentation, curves of maximal and mean velocities, as well as flow rates (mean velocity × segmented area for each phase), were derived (Figure 2d and 2e). To reduce the effect of noise, the maximal velocity was calculated for each phase as the average of pixels velocity values greater than 95% of the maximal velocity within the segmented ROI. The PC series acquired for transmitral flow analysis were used for aortic orifice delineation, resulting in ejection flow rate curves. However, because of the important obliquity between the aortic flow and the acquisition plane, aortic flow rate curves were only used for the estimation of temporal parameters. More precisely, the aortic flow rate curve was used to estimate the end of the ejection phase (Figure 2e). This time enabled the delimitation of the diastolic period, which was used for peak detection while analyzing transmitral blood flow maximal velocity and flow rate curves. The transmitral flow maximal velocity curve was used to estimate velocity-related parameters (early and late peak velocities E_MR _and A_MR_), by automatically detecting the two highest local peaks during the defined diastolic period (Figure 2d). For this detection, a temporal constraint, which consisted in requiring a minimal temporal distance of 1/6th of the cardiac cycle between E_MR _and A_MR _peaks, was first used to avoid detecting possible artifactual local peaks around E_MR _or A_MR_. Then, regardless of the magnitude, the peak that occurred first was defined as E_MR_, while the second was defined as A_MR_. Similar processing was applied on the transmitral flow rate curve to detect the peak filling rate (Ef_MR_, in ml/s) and the peak atrial rate (Af_MR_, in ml/s) (Figure 2e). The Ef_MR_/Af_MR _ratio, as well as the peak filling rate normalized by the filling volume Ef_MR_/FV_MR _(in s-1), were calculated. The filling volume (FV_MR_, in ml) was defined as the area under the transmitral flow rate curve comprised between the beginning and the end of the filling period, these times being defined as the intersection between the linear interpolation of the ascending and the descending slopes of the Ef_MR _and the Af_MR _waves and the time axis, respectively. Of note, flow rate curves, being estimated from the mean velocities, were preferred to maximal velocity curves for the estimation of temporal parameters because of their expected lower sensitivity to noise.

Finally, the isovolumetric relaxation time, IVRT_MR_, was estimated as the difference between the previously described beginning of the filling period and end of the ejection, and the deceleration time, DT_MR_, was calculated as the duration between the time to peak filling rate Ef_MR _and the end of the Ef_MR _wave (Figure 2e). The end of the Ef_MR _wave was estimated by linear interpolation of its descending slope. Of note, all linear interpolations of ascending and descending slopes were automatically performed on the part of the curve comprised between 40% and 70% of its maximal value, as previously presented in a study analyzing aortic velocity curves [17].

### Semi-automated detection of the myocardial velocity profiles

**Figure 3:**
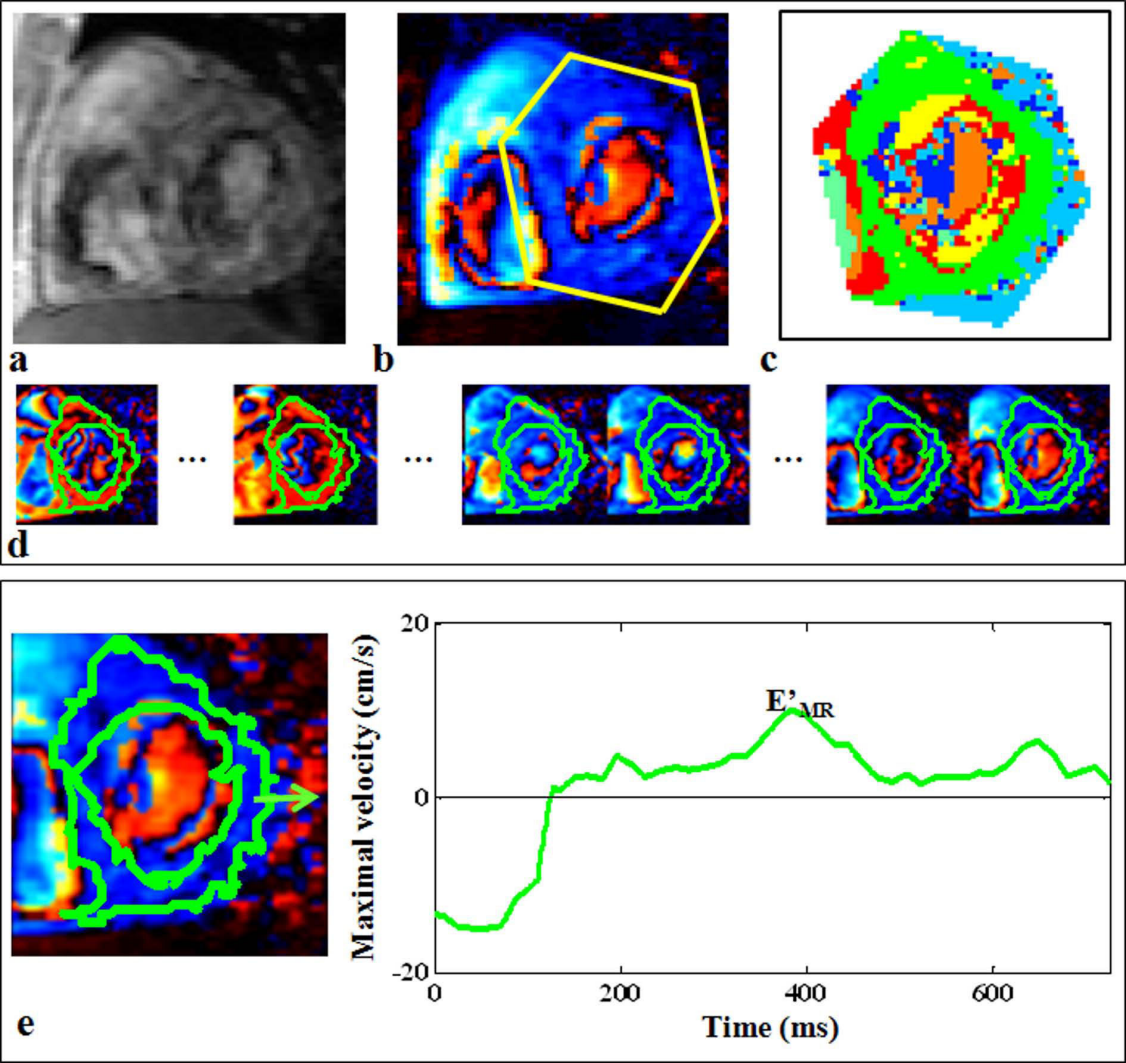
**Example of longitudinal tissue velocity evaluation from a myocardial PC dataset**. Top: detection of the myocardial cluster on a velocity-encoded image (b) using the k-means map (c), after manual drawing of a rough region of interest around the myocardium. The contours corresponding to the calculated myocardial cluster were superimposed on each cardiac phase, and shown on few selected phases (d) (see additional file 2: myocardiumVideo, video file corresponding to the whole cardiac cycle). Of note, the modulus image corresponding to the phase image (b) was shown (a) to highlight the difficulty of segmenting such images. Bottom: extraction of early peak diastolic longitudinal velocity (E'_MR_).

Similar to blood flow PC data, myocardial PC datasets contained a modulus (Figure 3a) and a velocity-encoded (Figure 3b) series. Because of the basal position of the imaging plane and of the low contrast between the myocardium and the neighbouring structures, myocardial detection on modulus images is even more challenging than on conventional cine MR images, especially for the epicardial wall (Figure 3a). Again, velocity images were preferred for the longitudinal motion analysis. However, the connectivity process was not adapted because of the bi-directional (up and down) longitudinal motion of the mitral annulus during a single cardiac cycle, which implies changes in velocity sign.

Accordingly, a classification based on the k-means algorithm [18] was applied on temporal velocity profiles of fixed pixels during a whole cardiac cycle, within a rough ROI manually drawn around the LV on a single phase (Figure 3b). This classification allowed isolating the biggest connected cluster, defined as the "myocardial" cluster (Figure 3c). We hypothesized that using the k-means classification while setting the number of clusters to seven would enable isolating the cluster corresponding to fixed pixels that remain within the myocardium during the entire cardiac cycle (Figure 3d) from neighbouring structures (background or LV cavity) or from clusters in which the longitudinal velocity profiles were distorted by the myocardial radial contraction. Maximal and mean velocity curves can be calculated from the obtained myocardial global cluster, as well as from standardized myocardial local segments (lateral, septal, inferior, and anterior segments) [19] (Figure 3e).

### Myocardial longitudinal velocities

The myocardial maximal longitudinal velocity curve, corresponding to the whole myocardium (Figure 3e), was used to derive the parameter E'_MR_, which was the highest local peak occurring first during the filling period. This peak velocity was used to estimate the conventional E_MR_/E'_MR _ratio.

### Evaluation of inter-operator variability

Since both flow segmentation and myocardial clustering required a manual initialization on a single phase, the inter-operator variability of our analysis in terms of blood flow segmentation, as well as functional velocity and flow rate parameters, was studied. For this evaluation, the whole process developed for flow and myocardial PC data analysis was repeated by two independent operators on a sub-group of 30 subjects including 20 controls and 10 patients.

### Statistical analysis

For both controls and patients groups, mean values and standard deviations of diastolic parameters, obtained from echocardiographic and CMR data, were reported. A non-parametric Mann-Whitney test was used to evaluate the significance of the differences between controls and patients functional parameters. A p value < 0.05 was considered as significant. In addition, Pearson correlation analysis was performed to compare CMR with Doppler echocardiography values. For both CMR and echocardiographic analyses, the ability of the calculated diastolic parameters to separate controls from patients, in terms of sensitivity, specificity, negative and positive predictive values (NPV and PPV) as well as the accuracy, was evaluated using a receiver operating characteristic (ROC) analysis to define optimal thresholds.

To evaluate inter-operator variability in terms of blood flow segmentation, the percentage of overlap between the two segmentations was calculated for each cardiac phase. The means and standard deviations of these percentages of overlap were calculated for the whole sub-group, on the diastolic period for the transmitral flow and on the systolic period for the aortic flow. Moreover, for both blood flow and myocardial parameters, inter-operator variability was calculated for each subject as the absolute difference of the repeated measurements in the percentage of their mean. These percentages were averaged on the whole sub-group.

## Results

All developments, including blood flow and myocardial detection, as well as the automated extraction of functional parameters from velocity and flow-rate curves, were integrated in a user-friendly interface developed on Matlab (Mathworks, Natick, MA, USA). This software was used to analyze PC data of the 53 subjects. For each subject, the processing time was less than 5 minutes, on a personal computer (CPU 2.67 GHz, 3 Gb RAM).

Blood flow segmentation was reproducible, as reflected by an averaged percentage of overlap between the segmentations performed by two independent operators of 99.7 ± 1.6% for the transmitral flow and 98.7 ± 7.1% for the aortic flow. Table 2 summarizes the inter-operator variability for both blood flow and myocardial functional parameters averaged over the sub-group of 30 subjects.

**Table 2:** Diastolic parameters measurement: inter-operator variability

	Inter-operator variability
E_MR_	0.14 ± 0.75%
A_MR_	0.11 ± 0.60%
E'_MR_	4.25 ± 5.89%
DT_MR_	1.96 ± 2.95%
Ef_MR_	0.14 ± 0.49%
Af_MR_	0.41 ± 1.44%
FV_MR_	0.34 ± 0.81%

Percentage of variation in diastolic parameters estimated by two independent operators averaged on a sub-group of 30 subjects

### Echocardiographic and CMR diastolic parameters

Table 3 summarizes mean values and standard deviations calculated for echocardiographic and CMR diastolic parameters on both controls and patients groups. Except for the IVRT_MR_, all echocardiographic and CMR diastolic functional parameters significantly varied in patients with aortic valve stenosis when compared to the controls.

**Table 3:** Echocardiographic and CMR diastolic parameters

	Controls	Patients	p value
**Echocardiographic measurements**			
			
E_US_/A_US_	1.39 ± 0.60	0.76 ± 0.27	<0.0001
DT_US _(ms)	180 ± 56	261 ± 59	0.0001
E'_US _(cm/s)	15.7 ± 4.2	8.0 ± 2.5	<0.0001
E_US_/E'_US_	5.3 ± 1.8	11.8 ± 7.6	<0.0001

**CMR measurements**			
			
E_MR_/A_MR_	1.33 ± 0.40	0.74 ± 0.27	<0.0001
Ef_MR_/Af_MR_	1.44 ± 0.58	0.54 ± 0.23	<0.0001
Ef_MR_/FV_MR _(s^-1^)	4.26 ± 0.93	2.55 ± 0.61	<0.0001
DT_MR _(ms)	185 ± 35	260 ± 40	<0.0001
E'_MR _(cm/s)	11.3 ± 3.5	7.3 ± 1.6	<0.0002
E_MR_/E'_MR_	5.3 ± 1.3	8.2 ± 2.5	<0.0002
IVRT_MR _(ms)	78 ± 29	94 ± 35	0.1

Mean values and standard deviations of the diastolic parameters calculated for controls and patients. Statistical significance of changes in diastolic parameters between controls and patients is provided

**Figure 4:**
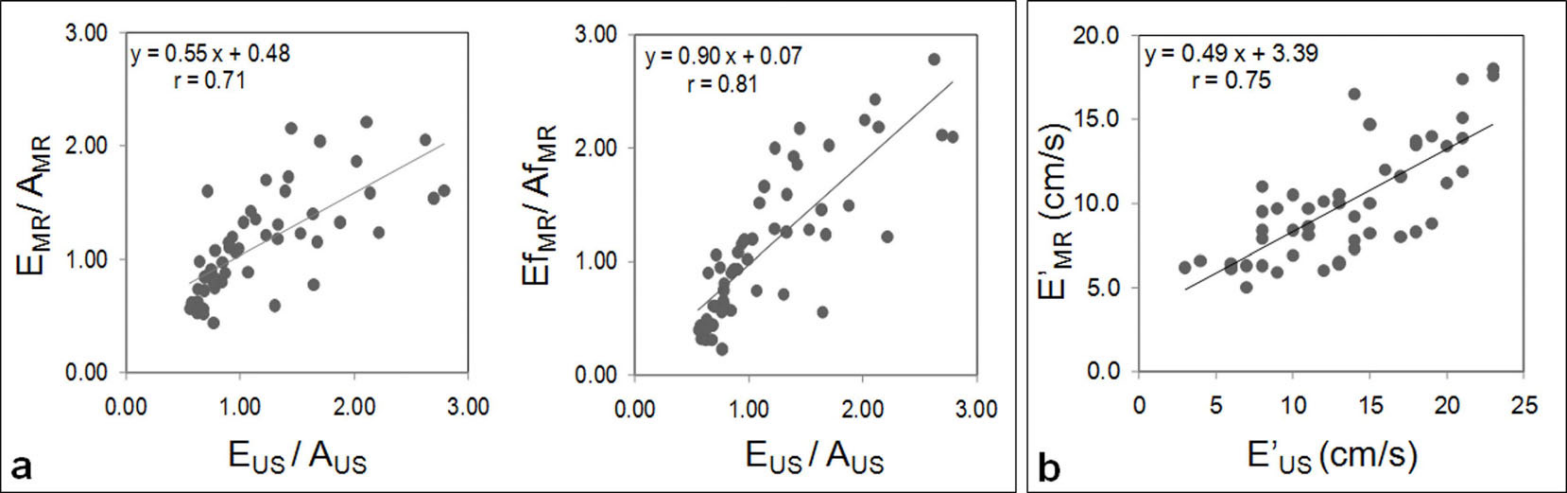
**Comparison between echocardiographic and CMR early to late peak ratios and mitral annulus peak longitudinal velocities**. Panel a: comparison of the CMR velocity (E_MR_/A_MR_) and flow rate (Ef_MR_/Af_MR_) ratios against the echocardiographic velocity ratio (E_US_/A_US_). Panel b: comparison between the mitral annulus peak longitudinal velocities estimated from echocardiographic data (E'_US_) and CMR data (E'_MR_).

A stronger correlation and a slope closer to 1 were found for the comparison between the echocardiographic E_US_/A_US _and the CMR flow rate-related Ef_MR_/Af_MR _than for the comparison with the CMR velocity-related E_MR_/A_MR _(Figure 4a). In addition, although the CMR mitral annulus longitudinal velocities E'_MR _were lower than echocardiographic values E'_US_, a good correlation was found between these two velocities (Figure 4b).

**Figure 5:**
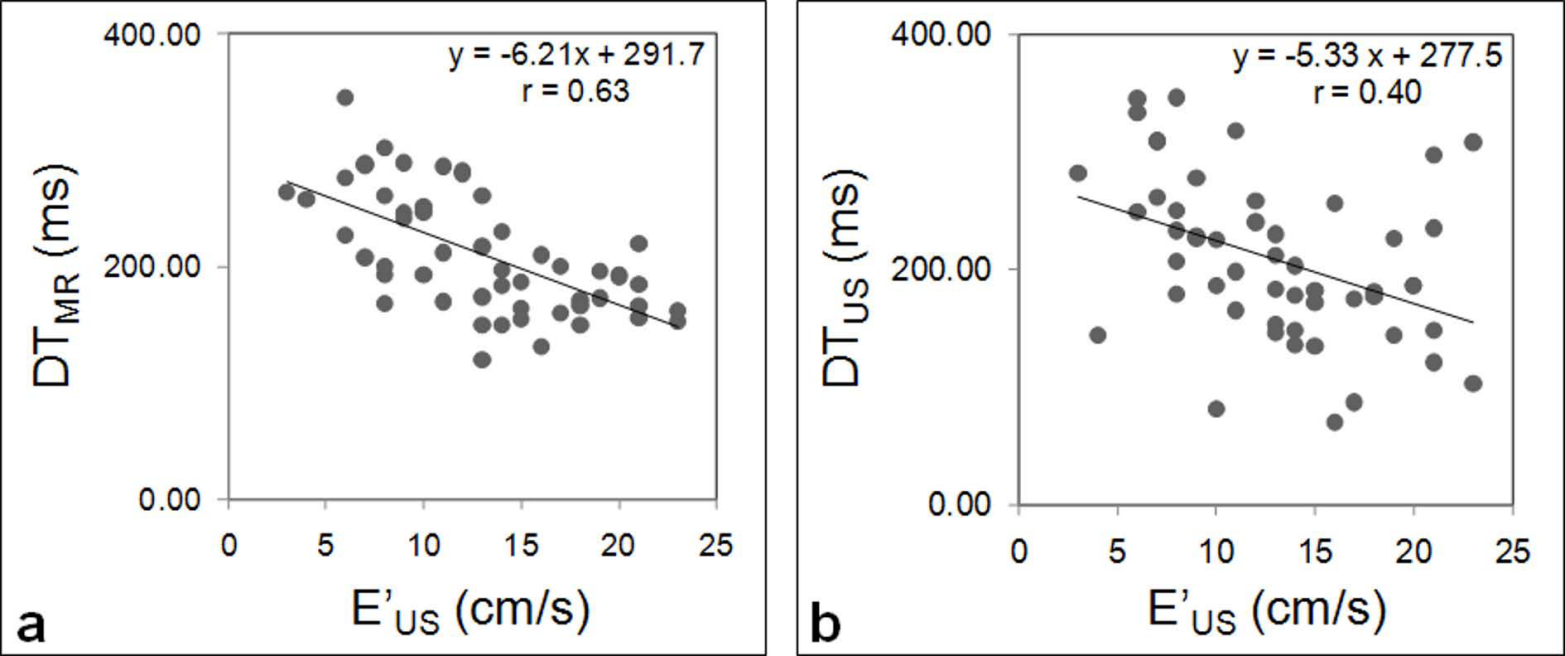
**Comparison of the echocardiographic and the CMR deceleration times against the echocardiographic mitral annulus peak longitudinal velocity**. Panel a: comparison between the echocardiographic mitral annulus peak longitudinal velocity (E'_US_) and the CMR deceleration time (DT_MR_). Panel b: comparison between the echocardiographic mitral annulus peak longitudinal velocity (E'_US_) and the echocardiographic deceleration time (DT_US_).

A fair correlation was found between echocardiographic and CMR deceleration times DT_US _and DT_MR _(r = 0.56). However, a stronger relationship was found between the echocardiographic mitral annulus longitudinal velocities E'_US _and the CMR deceleration time, DT_MR_, (Figure 5a) (r = 0.63, p < 0.0001) than for the comparison between the echocardiographic mitral annulus longitudinal velocities and the echocardiographic deceleration time, DT_US _(Figure 5b) (r = 0.40, p = 0.003).

### Abnormality detection

Table 4 summarizes the values of sensibility, specificity, negative and positive predictive values, and the overall accuracy obtained from the ROC analysis performed for each echocardiographic and CMR diastolic parameter on the 35 controls and the 18 patients with aortic valve stenosis. CMR diastolic parameters enabled abnormality detection with equivalent accuracy than the well established echocardiographic parameters. Indeed, all CMR parameters characterized the LV abnormality with a sensitivity and specificity above 0.80 and an accuracy above 0.85.

**Table 4:** Ability of echocardiographic and CMR diastolic parameters to separate controls from patients

	Sensitivity	Specificity	NPV	PPV	Accuracy	ROC threshold	AUC
**Echocardiographic measurements**							
							
E_US_/A_US_	0.78	0.94	0.88	0.89	0.89	0.77	0.89
DT_US _(ms)	0.83	0.76	0.65	0.90	0.79	212	0.84
E_US_/E'_US_	0.89	0.83	0.73	0.94	0.85	6.23	0.88

**CMR measurements**							
							
E_MR_/A_MR_	0.83	0.89	0.79	0.91	0.87	0.86	0.91
Ef_MR_/Af_MR_	0.83	0.91	0.83	0.91	0.89	0.71	0.95
Ef_MR_/FV_MR _(s^-1^)	0.89	0.91	0.84	0.94	0.91	3.17	0.95
DT_MR _(ms)	0.94	0.80	0.71	0.97	0.85	200	0.92
E_MR_/E'_MR_	0.80	0.87	0.75	0.90	0.85	6.5	0.84

Results of the receiver operating characteristic (ROC) analysis performed to calculate the sensitivity, specificity, negative and positive predictive values (NPV and PPV) and accuracy of each parameter. The ROC threshold and the area under the ROC curve (AUC) are provided

## Discussion

The early diagnosis of diastolic dysfunction has an important prognostic value and may impact the management strategy and the follow-up of patients with incipient heart failure. Although CMR is known as the modality of choice for the evaluation of global LV function [20,21], systolic function and myocardial viability [22,23], Doppler echocardiography remains the clinical reference for the evaluation of diastolic dysfunction [4,24,25]. Several CMR studies, based on volume variation curves extracted from cine images [26-29] or on velocity and flow rate curves extracted from PC images [8-15], reported capabilities of this modality for the assessment of diastolic function. However, despite these methodological developments and the recent technological improvements in PC-CMR sequences, the use of CMR in clinical evaluation of diastolic function remains limited because of the lack of automated methods designed for the analysis of PC images. Indeed, most of the PC-CMR studies previously presented in the literature were based on manual positioning of ROIs on each phase of the cardiac cycle [8-15]. This manual positioning is time-consuming [30] and subjective [8], leading to inter- and intra-operator variability, as reflected by the previously reported variability coefficient of 10% [31]. Accordingly, our primary goal was to minimize manual intervention to reduce variability and shorten the processing time. The final objective was to test the ability of the resulting CMR diastolic parameters to characterize LV diastolic dysfunction.

To achieve this aim, we first developed a connectivity-based technique for a semi-automated segmentation of the transmitral and the aortic flows patterns on blood velocity PC series. Because of the connectivity property, our technique is not related to the geometrical shape of the flow, which is an important feature of our technique. Thanks to this property, our segmentation method can be easily used for the delineation of various flow patterns, such as LV and right ventricular flows. In the present study, this segmentation was successfully used on the transmitral and the aortic flows of 53 subjects and was shown to be reproducible in a sub-group of 30 subjects, in terms of area overlap and functional parameters. The combination of this robust segmentation with an automated analysis of the derived velocity and flow rate curves enabled the estimation of consistent diastolic parameters. Indeed, despite the underestimation of velocity values, the comparison between the CMR and the echocardiographic E/A ratio revealed a good correlation. This correlation was higher and the slope of the linear interpolation between the CMR and the echocardiographic measurements was closer to one when considering the flow rate curves for the estimation of this ratio (Ef_MR_/Af_MR_). This finding might be related to the fact that flow rates are less sensitive to the shape of the velocity profile and to the slight mismatch between the acquisition plane and the true perpendicular to the transmitral flow.

In addition, the proposed flow rate-related parameters Ef_MR_/Af_MR _and Ef_MR_/FV_MR _resulted in a higher accuracy than the other CMR parameters, when used for LV diastolic dysfunction characterization (Table 4). Also, the CMR deceleration time estimated from the flow rate curve was more sensitive and more accurate than the echocardiographic deceleration time for the separation between controls and patients. The slight superiority of the flow rate-related parameters can be explained by the fact that flow rate curves are less sensitive to data noise than conventional maximal velocity curves, since they are estimated from the averaged velocity throughout the blood flow surface.

Secondly, a clustering technique [18] was used for an automated classification of velocity profiles from tissue velocity PC-CMR data. It enabled isolating the myocardial cluster and the corresponding maximal velocity curve during the cardiac cycle. To the best of our knowledge, the estimation of myocardial longitudinal velocities in the setting of diastolic function was previously presented in only few PC-CMR studies [8,11,13] and the positioning of myocardial ROIs was always done manually. In the present study, the only manual intervention was the positioning of a rough ROI around the LV, resulting in a very small inter-operator variability of the myocardial annular early peak longitudinal velocity E'_MR _(4.25 ± 5.89%). This variability was significantly lower than the 10% variability previously reported in a CMR study [31]. Of note, inter-operator variability of our CMR evaluation is also significantly lower than those previously reported in echocardiographic studies [25,32].

The comparison between CMR and echocardiographic mitral annulus longitudinal peak velocity resulted in a higher coefficient of correlation than r = 0.49 presented in a previous CMR study [8]. However, our CMR velocities were lower than the echocardiographic values. This might be due to the fact that Doppler values are derived from the envelop of the spectrum and to the difference in temporal resolution between the two techniques. Despite this underestimation, our CMR longitudinal velocity was significantly reduced in patients with severe aortic valve stenosis and the resulting ratio E_MR_/E'_MR _characterized LV dysfunction with a lower sensitivity than the echocardiographic ratio, but a higher specificity and an equivalent accuracy.

The differences in diastolic parameters found between echocardiography and CMR can be explained by the differences in imaging principles of the two techniques, including the difficulties of plane or beam positioning, but also by technical limitations inherent to the CMR acquisitions. These limitations included the limited temporal resolution of PC-CMR imaging as opposed to Doppler echocardiography, and the presence of phase offset errors, which were not corrected in the present study but were minimized using a 50% rectangular field of view centred on the mitral annulus. Alternatively, these errors can be corrected using techniques presented in previous studies [33-35]. Despite these technical limitations, high correlations were found between CMR and echocardiographic parameters and, more importantly, PC-CMR parameters were able to characterize LV diastolic dysfunction with the same accuracy than the echocardiographic indices.

## Conclusions

Our semi-automated method was fast, reproducible and was successfully used on PC-CMR blood flow and myocardial data of 53 subjects, including controls and patients with severe aortic valve stenosis and a preserved ejection fraction. This application enabled the estimation of velocity and flow rate-related diastolic parameters, which were highly correlated with echocardiographic measurements. In addition, significant differences were found between PC-CMR diastolic parameters estimated in controls and in patients with aortic valve stenosis, resulting in a high accuracy of the CMR characterization of LV diastolic dysfunction. Importantly, equivalent accuracy was found for both echocardiographic and CMR parameters, indicating a potential clinical usefulness of CMR for the evaluation of diastolic function, which however should be confirmed by additional studies performed on larger populations with subtle to severe diastolic dysfunction.

## Competing interests

The authors declare that they have no competing interests.

## Authors' contributions

EB and NK participated in the design of the study, in the technical developments, and in writing the manuscript. AR participated in the interpretation of the CMR data and in revising the manuscript. SCG, CD, MLa and LP participated in the acquisition and interpretation of echocardiographic data. MLe participated in data processing for the reproducibility study. ADC and AH participated in the technical developments. BD and EM participated in the design and coordination of the study. All authors read and participated in revising the manuscript. They all approved its final version.

## Supplementary Material

Additional file 1 **Example of transmitral and aortic flow detection on all phases of the cardiac cycle**. Beginning of the video (systolic phase): the contours corresponding to the aortic flow were superimposed on each colour-coded velocity image. End of the video (diastolic phase): the contours corresponding to the transmitral flow were superimposed on each colour-coded velocity image. Click here for file

Additional file 2 **Example of the superimposition of the myocardial cluster on all phases of the cardiac cycle**. The myocardial cluster defined by the k-means algorithm is superimposed on the colour-coded myocardial PC velocity images. Click here for file
